# Transfer Information Assessment in Diagnosis of Vasovagal Syncope Using Transfer Entropy

**DOI:** 10.3390/e21040347

**Published:** 2019-03-29

**Authors:** Katarzyna Buszko, Agnieszka Piątkowska, Edward Koźluk, Tomasz Fabiszak, Grzegorz Opolski

**Affiliations:** 1 Department of Theoretical Foundations of Bio-Medical Science and Medical Informatics, Collegium Medicum, Nicolaus Copernicus University, 85-067 Bydgoszcz, Poland; 2 Department of Emergency Medicine, Wroclaw Medical University, 50-556 Wroclaw, Poland; 3 1st Chair and Department of Cardiology, Medical University of Warsaw, 02-097 Warsaw, Poland; 4 Department of Cardiology and Internal Diseases, Collegium Medicum, Nicolaus Copernicus University, 85-067 Bydgoszcz, Poland

**Keywords:** transfer entropy, vasovagal syndrome, head up tilt test

## Abstract

The paper presents an application of Transfer Entropy (TE) to the analysis of information transfer between biosignals (heart rate expressed as R-R intervals (RRI), blood pressure (sBP, dBP) and stroke volume (SV)) measured during head up tilt testing (HUTT) in patients with suspected vasovagal syndrome. The study group comprised of 80 patients who were divided into two groups: the HUTT(+) group consisting of 57 patients who developed syncope during the passive phase of the test and HUTT(−) group consisting of 23 patients who had a negative result of the passive phase and experienced syncope after provocation with nitroglycerin. In both groups the information transfer depends on the phase of the tilt test. In supine position the highest transfer occurred between driver RRI and other components. In upright position it is the driver sBP that plays the crucial role. The pre-syncope phase features the highest information transfer from driver SV to blood pressure components. In each group the comparisons of TE between different phases of HUT test showed significant differences for RRI and SV as drivers.

## 1. Introduction

Vasovagal syncope (VVS) belongs to the neuro-cardiogenic type of syncope and it usually results in a fall. It is characterized by rapid onset, short duration, and spontaneous complete recovery [1]. It is defined as a transient loss of consciousness (TLOC) due to cerebral hypoperfusion [1]. Vasovagal syncope is often triggered by emotions or strong stress. It can also occur during a vein puncture or prolonged upright tilt, particularly in stuffy rooms [2]. However, the main causes and pathophysiological mechanisms of this type of syncope remain unknown. In general, there are two physiological theories that attempt to explain the mechanism of vasovagal syncope. Van Lieshout et al. proposed a central theory that assumes that the reflex is triggered by activation of the cortical-subcortical centers, releasing necrohormones and neurotransmitters. This in turn leads to the bradycardia-hypotension reflex in response to factors such as pain, fear or emotions [3]. Oberg and Thoren proposed a peripheral theory which suggests that the reflex is triggered by stimulation of the cardiac mechanoreceptors in the left ventricle, cardiac atria and aortic arch as well as peripheral vascular chemoreceptors due to maintaining the upright body position for prolonged periods of time [4]. The common link of these theories is that the actual cause of syncope is central hypovolemia caused by deposition of blood pooling in lower extremities and skeletal muscles, resulting in a drop of blood pressure, bradycardia, and a drop in skeletal muscle tone. The two theories presented above as well as other less popular theories, do not fully explain the pathophysiological mechanism of vasovagal syndrome. Because of that, new and more advanced methods of analysis of syncope should be tested to better understand its mechanisms. We propose transfer entropy as a nonlinear method for investigating information transfer between physiological components that interact before and during syncope. As mentioned above, the mechanism of the interaction is not fully known and understood. We believe that the choice of the transfer entropy as a tool for analysis of syncope would bring us closer to understanding the mechanism of VVS. We would like to evaluate if changes of information transfer during a Head Up Tilt Test (HUTT) can shed some light on pathophysiological mechanism of VVS.

The choice of transfer entropy can be backed up by two reasons. The first relates to the properties of the method: it does not rely on any model and it needs neither the assumptions on the nature of the signals nor the analyzed interactions between them. The second one relates to the fact that transfer entropy was successfully applied in analysis of transfer of information in physiological systems [5,6,7,8,9,10,11,12,13,14,15,16].

Typically, diagnosing VVS requires the HUTT to be performed. During the test three types of physiological signals are continuously recorded: electrocardiogram (ECG), systolic and diastolic blood pressure (sBP and dBP). Sometimes, additionally the impedance cardiography (ICG) is recorded too. Our calculations were made on the basis of the latter of the signals recorded in different phases of the test. We also took into consideration the type of provocation of vasovagal reaction, separately analyzing the group of patients that experienced syncope in the passive phase of the HUT test and the group with a positive HUT test outcome only after pharmacological provocation with nitroglycerine (NTG). The HUT test is performed according to the strictly defined protocol. In fact, there is no single recommended protocol. Westminster and Italian are the most popular. All protocols have two common features: they are composed of three phases (supine position, tilt and pre-syncope) and the duration of the test is long (even more than 90 min). Despite the test is painless, it is inconvenient for the patients due to its long duration and the inevitability of syncope occurrence. The possibility of prediction of passive HUT test outcome in the supine phase of the test or in early post-tilting allows to cut the tilt test duration (even to 30 min) and omit the feeling of syncope. The reduction of the test duration allows also to reduce the costs of the examination and makes possible to perform more tests per day. Therefore the main objective of this paper is to evaluate if the Transfer Entropy of simultaneously measured RRI, sBP, dBP and SV can be a useful tool for prediction of a vasovagal syndrome during HUTT. In our previous investigation we attempted to find entropy measure, which would be used as a tool of prediction of the HUT test outcome. However, the applied entropy measures analyzed each measured parameter separately. Against this background, the transfer entropy allows to investigate the relation between the parameters during the test. Moreover the algorithm of calculation of Transfer Entropy is based on assessment of Conditional Entropy, which has been calculated in the previous investigation and it turned out to be a useful tool in passive HUT test prediction [17]. The features of TE mentioned above motivated us to explore the TE in diagnosing of VVS.

We based our analysis on multiple statistical tests, therefore the presented investigation should be treated as an exploratory research. The results of statistical tests may indicate the correctness of the hypotheses formulated by us, but they cannot be treated as final results. The paper comprises four sections: i.e., an introduction, the characteristics of materials and methods, research results and discussion.

## 2. Materials and Methods

### 2.1. Study Group

We conducted a retrospective, single-center analysis based on data obtained in the period between 2005 and 2013. The data were collected by the first Chair and Department of Cardiology and The Cardiology Outpatient Clinic at the Medical University of Warsaw. The study was compliant with the Declaration of Helsinki. Prior to enrolment in the study, each patient gave their informed consent for data acquisition and their use for scientific purposes.

The study was performed on a database including 230 patients with a history of prior syncope. Only patients suffering from neuro-cardiogenic syncope were considered eligible for the study. Patients with brain or heart diseases were excluded from the study. The final group consisted of 80 patients. All participants underwent a Westminster Protocol guided HUTT [1] for diagnosis of VVS.

For classification of VVS theVasovagal Syncope International Study (VASIS) criteria were applied [18]. The 57 patients who experienced syncope in the passive phase of the test were labelled as the HUTT(+) group. The remaining 23 patients who remained uneventful during the passive phase of the test and developed syncope only after being pharmacologically provoked with nitroglycerin were described as HUTT(−). The baseline characteristics of the patients of both groups are presented in Table 1.

We obtained approval for our research from The Ethics Committee of The Medical University of Warsaw, Poland (approval number: AKBE/51/2018). 

### 2.2. Data Collections

The physical examination used for diagnosing of syncope was the HUTT, usually performed with a Task Force Monitor (TFM) [19,20,21]. The TFM consists of two components. The first one is a tilt table with a footboard and abdominal straps. The second component comprises devices for continuous monitoring of continuous monitoring of electrocardiogram (ECG), impedance cardiography (ICG) and blood pressure. The procedure requires the patient to lie on the table which is tilted to different angles ranging from 60 to 90 degrees. The patients were fasting prior to the test. The day before the test they also had to refrain from consumption of coffee and alcohol. If used, beta-blockers were discontinued two days prior to the test. Other antihypertensive drugs were continued without any regimen modifications. The tests were performed in the morning in a dimly lit, quiet room, at a controlled temperature of 23–24 °C. A modified Westminster protocol was used for the testing (1): 20-min rest in the supine position, then a rapid tilt to 60 degree (within 5 s); next, the patient remained upright for 45 min or until syncope or pre-syncope occurred. If no syncope was induced by this time, 0.4 mg nitroglycerin (NTG) was administered sublingually to aid in syncope provocation and the test itself was prolonged by additional 20 min [22].

There were three patients with contraindications to NTG administration due to a significant drop in the sBP (below 100 mmHg at the end of the passive phase) who did not experience syncope after the initial 45 min. In those cases we prolonged the test by another 15 min, which resulted in occurrence of syncope in all three cases.

In the passive phase of the test the average time of from the tilt to syncope onset was 20 min and ranged from 3 up to 52 min. When pharmacological provocation had to be implemented, we observed that syncope occurred in a time range between 2 and 6.8 min, with the average time to syncope onset of 3.8 min from the administration of NTG.

### 2.3. Data Analysis and Statistical Methods

**Figure 1:**
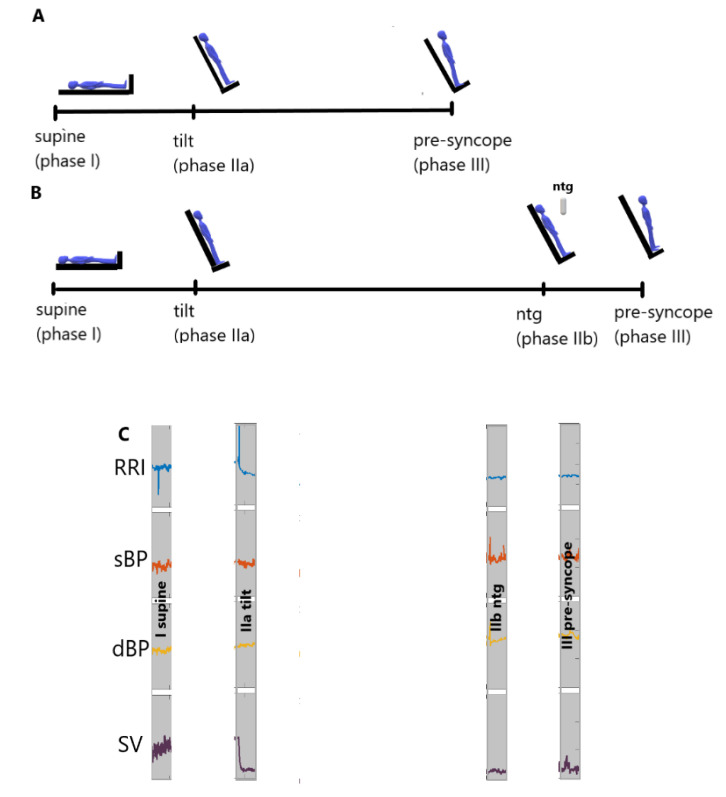
The scheme of the head up tilt test: (**A**) passive test (**B**) test with nitroglycerine provocation (**C**) the windows of recorded data used for Transfer Entropy calculations for HUTT(-) group.

We recorded three physiological biosignals during HUT testing process: high resolution ECG (2-channels with a sampling frequency of 1000 Hz), sBP and dBP measured continuously and ICG. Then, we conducted an analysis using: RRI obtained from the ECG recordings, SV taken from the ICG curve and blood pressure (sBP, dBP). Beat-to-beat recordings were used by the TFM system to obtain the signals. All signals obtained from the TFM were verified by a clinician. We excluded ectopic values, however they accounted for less than 5%. The scheme of measurements during the HUT testing is presented in Figure 1. We chose four 250-beats intervals of data (“windows”) in each of the following four stages of the HUTT: supine (phase I), tilt (phase IIa), NTG (phase IIb) and pre-syncope (phase III). The analysis of the data inside the windows was performed on the series normalized to a zero mean and the variance amounted to one.

Transfer entropy (TE (driver-target)) was determined separately for the HUTT(+) and HUTT(−) groups, for each possible pair of the variables: RRI, sBP, dBP and SV conditioned to the three other in each phase of the test. Due to the lack of normal distribution and lack of variance equality, for statistical analysis of the data regarding TE descriptive statistics (mean ± std), median with IQR and nonparametric tests were used. The comparison of entropies between the data in windows was performed using the Friedman test and the post-hoc Dunn test. The tests included multiple comparison correction and the Bonfferoni correction was used for the correction of desired significance level α = 0.05. For the HUTT(+) group it was 0.016 and for the HUTT(-) it was 0.008.

For comparisons of entropies between the HUTT(+) and HUTT(−) groups within each HUTT phase the Mann-Whitney test was used. The *p*-values were also adjusted for multiple comparisons using Bonfferoni correction. Statistical calculations were performed using the Matlab 2017b (MATLAB and Statistics Toolbox Release 2017, The MathWorks Inc., Natick, MA, USA) and Statistica 13.1 (StatSoft Inc., Tulsa, OK, USA).

Transfer Entropy calculations were performed with the algorithms proposed by Montalto et al. (MuTE: available on website [23]) and described in [5]. The parameters and the method applied for TE calculations were chosen in line with the recommendation for cardiovascular data presented in [5]. We used the estimation of TE based on binning estimator (BIN) with the embeding vectors determined by non-uniform embedding estimator (NUE) [5], assuming the following parameters of the method: time lag 1–5 and quantization level equal 6. A brief description of Transfer Entropy is presented in the next subsection.

### 2.4. Transfer Entropy

The idea of Transfer entropy was proposed by Schreiber in 2000 [1]. Since that time it has been widely used as a tool for detecting information transfer between joint processes in numerous fields. In our investigation we applied the algorithm of TE proposed by A. Montalto et.al [2]. The algorithm and MatLab toolbox for calculating TE were described in detail in [2]. Here, we briefly recall the main idea of Transfer entropy and the procedure of its calculation.

Let us assume X as a system of source information and Υ as a destination system in a set of M interacting dynamical systems. The remaining systems of M are described as $Z={\left\{Z^{k}\right\}}_{k=1,\ldots ,M-2}$. After sampling procedure of stochastic processes X, Y, **Z** describing the mentioned systems, we introduce the variables $X_{n},Y_{n},Z_{n}$ describing the processes in present time n. Consequently the past processes of X, Y and **Z** are represented by: $X_{n}^{-}=\left[X_{n-1}X_{n-2}\ldots \right]$, $Y_{n}^{-}=\left[Y_{n-1}Y_{n-2}\ldots \right]$ and $Z_{n}^{-}=\left[Z_{n-1}Z_{n-2}\ldots \right]$. The Transfer Entropy (TE) from X to Y conditioned to **Z** is defined as follows:

(1) $$TE_{X\to Y|Z}=\sum p\left(Y_{n},Y_{n}^{-},X_{n}^{-},Z_{n}^{-}\right)\log\frac{p\left(Y_{n}|Y_{n}^{-},X_{n}^{-},Z_{n}^{-}\right)}{p\left(Y_{n}|Y_{n}^{-},Z_{n}^{-}\right)}$$

In terms of Conditional Entropy (CE) the formula (1) can be written as:

(2) $$TE_{X\to Y|Z}=CE\left(Y_{n}|Y_{n}^{-},Z_{n}^{-}\right)-CE\left(Y_{n}|Y_{n}^{-},X_{n}^{-},Z_{n}^{-}\right)$$

In terms of Shannon Entropy (ShE) the formula (1) can be written as:

(3) $$TE_{X\to Y|Z}=ShE\left(Y_{n},Y_{n}^{-},Z_{n}^{-}\right)-ShE\left(Y_{n}^{-},Z_{n}^{-}\right)-ShE\left(Y_{n},Y_{n}^{-},X_{n}^{-},Z_{n}^{-}\right)+ShE\left(Y_{n}^{-},X_{n}^{-},Z_{n}^{-}\right)$$

The main problems of the calculation of Transfer Entropy are: reconstruction of the past states of the system and evaluation of TE. Let us assume $V=\left[V_{n}^{Y},V_{n}^{X},V_{n}^{Z}\right]$ as a vector containing the most significant past variables that are able to explain the present state of destination process Y. Montalto et al. [2] proposed two method of the vector V determination: uniform embedding (UE) and non-uniform embedding (NUE). In our calculation we applied the NUE method. The main idea of NUE is based on progressive selection the most informative lagged variables of X, Y and **Z** for the target $Y_{n}$. In the final step of the procedure the vector V includes the most relevant components of $Y_{n}^{-},X_{n}^{-},Z_{n}^{-}$ for description of $Y_{n}$. The choice of such significant components is based on the test for candidate significance: the selected candidate is significant if the conditional mutual information between target variable and the candidate is above the 95-th percentile of its null distribution. As a result, the embedding vector is composed of statistically significant components described the target. The details of the procedure of building optimal embedding vector was described and discussed in details in [2]. Montalto et al. [2] proposed three types of estimation of TE: linear estimator (LIN), classical binning estimator (BIN) and k-nearest neighbor techniques (NN). According to the recommendation included in the paper [2] we used the BIN estimator. In this estimation the uniform quantization of the time series is preformed and the entropy is approximated by probability of visitation of the quantized states. It can be described by Equation (2):

(4) $$H\left(V_{\xi }\right)=-\underset{V_{\xi }\epsilon A^{d}}{\sum }p\left(V_{\xi }\right)\log p\left(V_{\xi }\right)$$

where ξ denotes quantization levels, $V_{\xi }$ is the quantized vector. All vectors V, that fall in the same hypercube of size r are associated with $V_{\xi }$. The $\xi^{d}$ disjoint hypercubes are created during the uniform quantization of embedding vectors of dimension d.

The significance of TE is calculated in comparison with its null distribution performed on TE computed on replications of the original time series. In each replication the randomly selected lag was used to create time-shifted series from the source series [2]. The details of the algorithm are presented in [2]. The analysis of TE allows to specify which parameter of the HUT test plays essential role in information transfer in each phase and to which most of the information is transferred (the highest value of TE (driver-target)). This knowledge allows us to identify which component of cardiovascular mechanism, being the main effector of autonomic nervous system, controls the development of vasovagal syndrome occurrence during the test.

## 3. Results

### 3.1. Comparisons of Transfer Entropy between Different Tilt Test Phases for HUTT(+) and HUTT(-) Group

We calculated Transfer Entropy (TE (driver-target) for RRI, sBP, dBP and SV recorded during consecutive HUTT phases (Figure 1). The descriptive statistics of TE, i.e., mean ± standard deviation (std) for the HUTT (+) and HUTT (−) groups are presented in Table 2 and Table 3 respectively.

**Figure 2:**
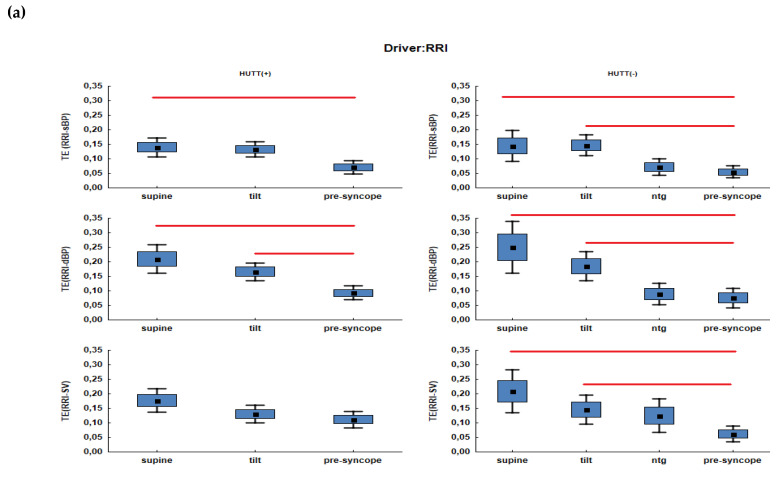

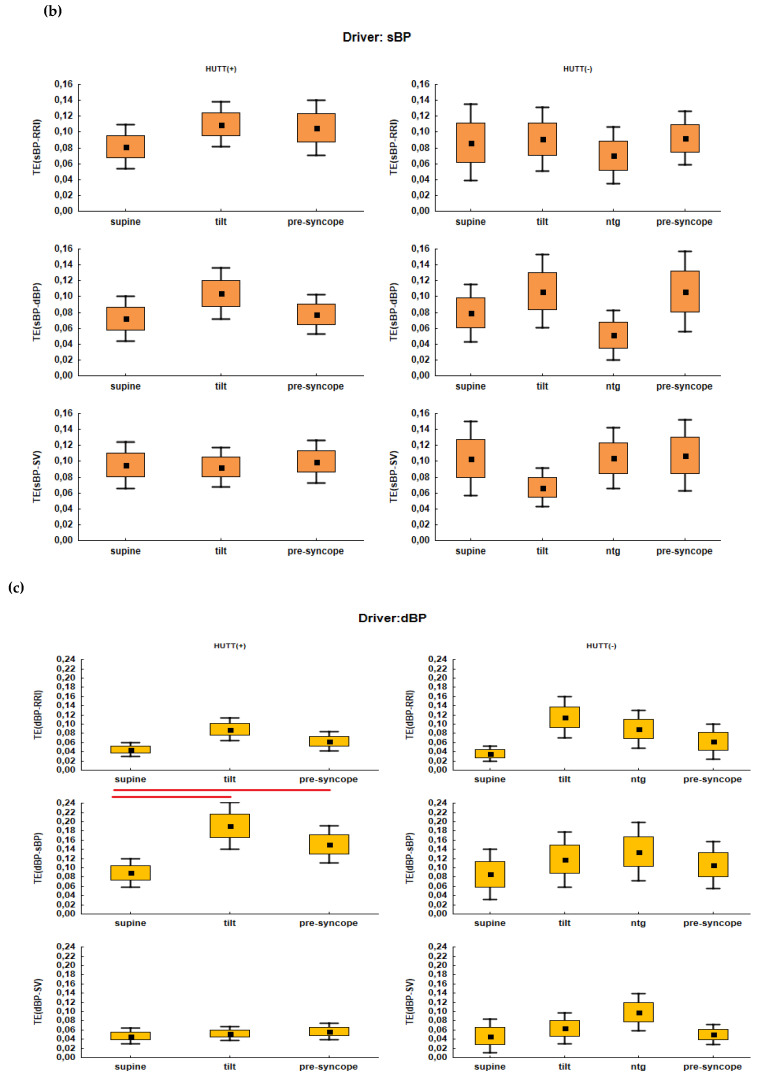

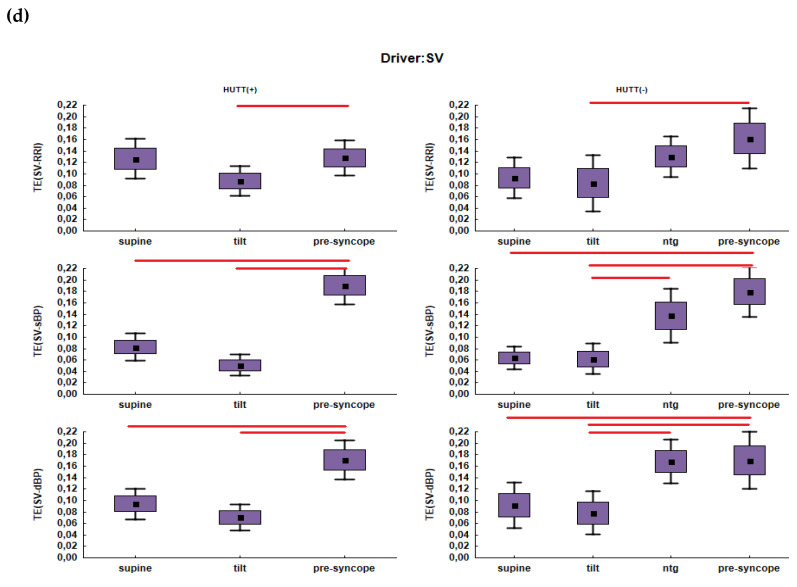
Transfer Entropy (TE) in consecutive HUTT phases for the HUTT(+) (on the left) and HUTT(−) group (on the right) for the following drivers: (**a**) RRI, (**b**) sBP, (**c**) dBP, (**d**) SV.

Figure 2a–d show plots of TE (driver-target) for each group and for all possible combinations of drivers and targets.

We marked the plots of TE calculated for driver RRI in blue, for driver sBP in orange, for driver dBP in yellow and for driver SV in purple. The squares included in the plots depict means, the box refers to the standard error (SEM) while the whiskers refer to confidence intervals. Significant results of multicomparison tests are marked as red lines.

### 3.2. Comparisons of TE between HUTT(-) versus HUTT(+) Group in Individual Phases of HUTT

We compared TE of two groups of patients in three phases of the HUTT common for both groups (phase I, IIa and III), using the Mann–Whitney test. Table 4 presents the results of this comparison.

**Figure 3:**
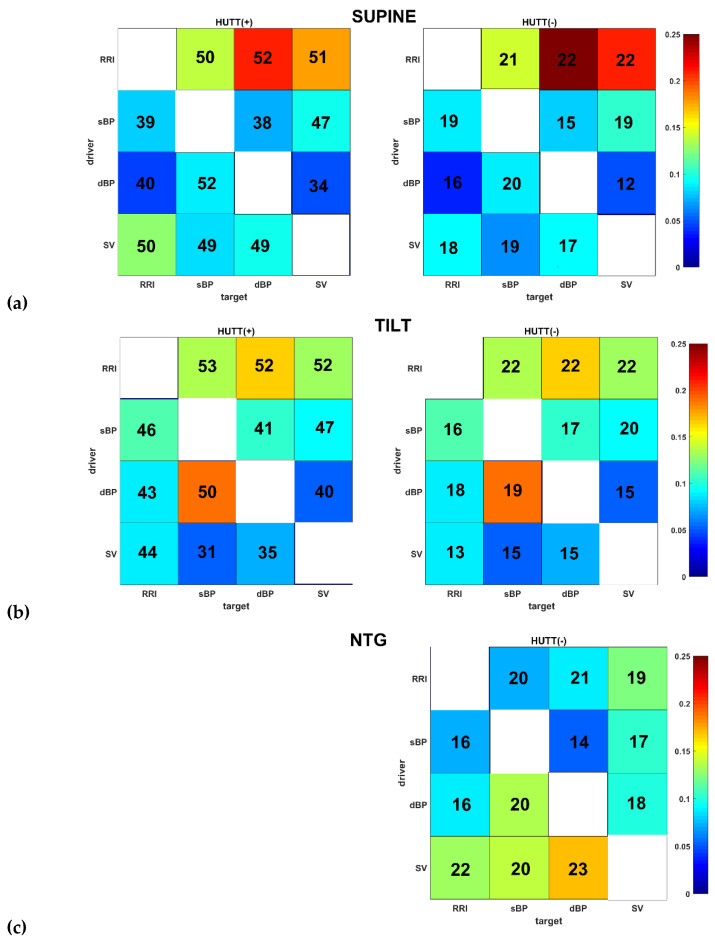

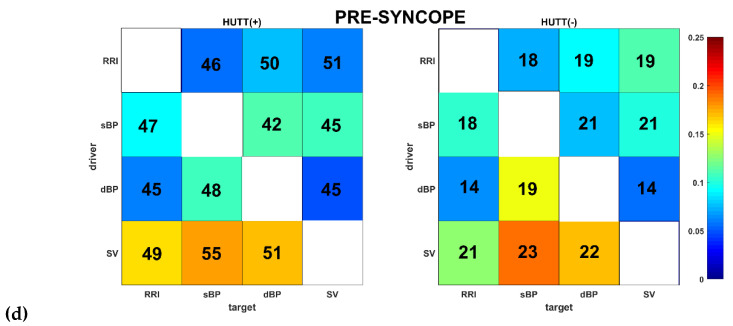
TE matrix representation for HUTT(+)(left side, *n* = 57) and HUTT(-) (right side, *n* = 23) groups in the phases of HUTT: (**a**) supine-I (**b**) tilt-IIa (**c**) ntg-IIb (**d**) pre-syncope-III. The colors indicate the magnitude of TE averaged over the results obtained for all patients. The color-scale is the same for each matrix. The deep blue corresponds to TE equal approximately to 0 and the brown color corresponds to TE equal approximately to 0.25. The diagonal matrix elements were marked as white because they were excluded from the TE calculations. Each number expresses the number of significant links of corresponding pair driver-target.

Figure 3 (panels a–d) presents TE matrices separately for the HUTT(+) (left side) and HUTT(-) group (right side). Each element of the TE matrix represents average TE of the results obtained for all patients. The magnitude of TE is color-coded. Each number expresses the number of significant links of corresponding pair driver-target.

In each matrix the single element is a number of statistically significant TE (driver-target) obtained for the analyzed group of patients (in HUTT(+) group *n* = 57, in HUTT(-) group *n* = 23). The determination of significance of TE was described in Section 2.4.

## 4. Discussion

It is commonly accepted that physiological time series such as RR intervals and blood pressure and SV are complex and have a nonlinear nature, therefore the application of linear analysis is inappropriate. Researchers widely applied typical linear tools for analyzing synchrony and similarity between two signals. Traditionally the investigation under synchronization measurement between two signals is performed in frequency domain: the spectral analysis and further analysis of coherence between the signals. Due to the non-stationarity of the physiological time series the spectral estimators are often biased and inconsistent. Therefore spectral analysis is not suitable for the assessment of signals’ synchrony. Coherency as a measure based on linear correlation, used as a function of frequency, is also not suitable for non-stationary signals analyzing. Coherence measurement can only detect linear relations between the signals in frequency domain and is not useful for detecting nonlinear changes between them. Another popular method is phase-amplitude coupling (PAC) used especially by researchers in neuroscience. PAC allows to measure the interactions between neuronal oscillations and assess the modulation of phase of lower frequency oscillations on amplitude of the higher frequency oscillations [24].

In recent years the synchronization measurement based on entropy measures as a nonlinear approach became very popular. In physiological time series analysis such tools allow to analyze coupling between two signals and cross-prediction. Li et al. [25] found using multiscale multivariate fuzzy entropy (MMFE) that in patients with heart failure the coupling between heart rate variability (HRV) and diastolic period variability (DPV) is reduced in small and large scales in comparisons with healthy subjects. They also found that in healthy subjects the coupling is reduced with age in small scales [26]. In order to detect the synchronization of the bivariate time series there are also widely used: cross sample entropy (C-SampEn), cross fuzzy entropy (C-FuzzyEn) and cross fuzzy measure entropy (C-FuzzyMEn) [27,28,29,30,31]. The investigation under the measuring of synchronization in coupled cardiovascular time series performed by Liu et al. [27] showed that the three cross-entropy measures between RR intervals and pulse transit time (PTT) in patients with heart failure are lower than in healthy subjects. The mentioned nonlinear measures of synchronization are based on the entropies belonging to the family of Approximate Entropy proposed by Pinus [32]. However, the cross-entropy approach focuses only on two coupled signals. In our investigation we analyze four signals simultaneously measured during the HUT test. The application of Transfer Entropy allowed us to assess information transfer between two processes conditioned to others processes. We can also investigate nonlinear interactions in the analyzed system with a wide range of interaction delays. The transfer Entropy is based on the assessment of conditional entropy. In our previous investigations the Conditional Entropy occurred the only entropy measure, which has a prognostic value in predicting the HUT test. The entropy measures from the ApEn family (Approximate Entropy, Sample Entropy and Fuzzy Entropy) did not turn out good candidates for VVS prediction in a passive HUT test [17]. The two mentioned reasons induced us to choose the Transfer Entropy as a measure of the relation between the measured parameters during the HUT test.

Transfer was introduced by Schreiber in 2000 [33]. Since that time Transfer Entropy has been widely applied in analysis of physiological time series especially in neurophysiology and cardiovascular physiology [5,6,7,8,9,10,11,12,13,14,15,16,34]. In neurophysiology, calculations are performed primarily on the basis of EEG analysis. In cardiology, such analyses are conducted on heart rate (HR), blood pressure (BP), systolic arterial pressure (SAP), respiratory volume signal, cerebral blood flow velocity (CBFV) and muscle sympathetic nerve activity (MSNA) [13,35]. The research embraces various groups of patients, from healthy volunteers to patients suffering from: epilepsy, unexplained syncope, congestive heart failure, etc. [35,36,37]. Apart from direct analysis of continuously measured signals, some investigators performed calculations on modified variables such as spectral components of RRI (LF and HF) [37,38]. For example, Luo et al. [37] used TE to show the interaction between the sympathetic nervous system (SNS) and the parasympathetic nervous system (PNS) in patients with congestive heart failure (CHF) and healthy subject. They calculated TE(LF-HF) and TE(HF-LF) for both groups in order to quantify the information exchanged between SNS and PNS. In comparisons with healthy subject, CHF patients were observed to have increased TE.

We applied transfer entropy (TE) to assess the information transfer between four biosignals (RRI, sBP, dBP and SV) measured continuously during HUT testing performed in patients suffering from vasovagal syncope. In calculations of TE we observed:

- in supine position (phase I): TE was greater than 0 in both groups for all driver-target pairs. The highest values of TE were noted for RRI-dBP and RRI-SV (Figure 3a) and lightly lower TE was observed for the RRI-SV link.
- in response to the tilt (phase IIa): highest values of TE were also found in both groups for the RRI-dBP links and slightly lower for: RRI-sBP and RRI-SV (Figure 3b). In the HUTT(+) group we noted a high value of TE(dBP-sBP).
- in the phase IIb (nitroglycerin administration): HUTT(-) manifested the highest value of TE for the SV driver to the following targets: RRI, sBP and dBP (Figure 3c).
- in the pre-syncope phase (III): the highest information transfer in both groups was related to the SV driver in conjunction with RRI, sBP and dBP as targets (Figure 3d). In both groups, the highest was TE(SV-sBP) and slightly lower TE SV-dBP) and T (SV-RRI).

During the HUTT in supine position, the main information transfer is from RRI to other signals. This key role of RRI, however, was reduced after the tilt and the main information transfer was then from sBP to other signals (Figure 3b). This finding is not surprising, because of its obvious physiological interpretation [5,16,34] as during the transition from supine to upright position the sympathetic nervous system is activated while the parasympathetic nervous system deactivates. Consequently, it increases the baroreflex regulation. Porta et al. analyzed spontaneous baroreflex regulation in healthy subjects using information transfer. They evaluated the strength of the causal relation along baroreflex regulation during the tilt test. In their investigation they showed that indices based on transfer entropy are significantly related and complementary with traditional baroreflex indices [13,14]. Wejer et al. also used transfer entropy in analysis of coupling between cardiac and vascular systems in healthy subject during the head-up tilt test. They confirmed the essential role of baroreflex regulation in response to tilt and denoted switches in the system of drivers in consecutive phases of the HUTT. In the early stages of tilt, the vascular system plays the role of the driver, but gradually the cardiac system takes over and in the late tilt stage it is the vascular system take over again [15]. The assessment of cardiovascular regulation during head-up tilt with application of transfer entropy was also performed by Graff et al. [16]. They analyzed information transfer between heart rate and blood pressure in response to upright position during the HUT test in two groups of patients: healthy volunteers and patients who suffer from VVS. They calculated TE(RRI-sBP) and TE(sBP-RRI) and obtained similar values for both in supine position for the investigated groups of patients. In upright position they noted higher values of TE (sBP-RRI) in both groups and interpreted this finding as the reflection of baroreceptors activation. Moreover, they observed significantly lower TE(sBP-RRI) in VVS patients than in healthy subjects. In our analysis, no significant differences in TE(sBP-RRI) were found in consecutive phases of HUTT for the HUTT(+) as well as HUTT(-) group. We suppose that this finding is one of the features characteristic for VVS patients and it seems to be associated with the mechanism of VVS occurrence. The second interesting feature was observed in the pre-syncope phase. Unlike in phase I and II, in the pre-syncope phase, the main information transfer is from SV to others signals (Figure 3). We do not know what the physiological interpretation of this result is, but it shows that the role of SV in the vasovagal syndrome analysis is relevant. The classification of the type of vasovagal reaction is based then on the occurrence of changes in blood pressure and heart rate during the pre-syncope phase. Thus, the role of SV changes for vasovagal reaction is being highlighted. It means that the further research on VVS pathophysiological mechanism is required with inclusion of response of cardiac mechanoreceptors. The precise determination of the mechanism of syncope and the contribution of the cardiac component is essential for the risk stratification of the patient.

We expect that further explanation of the VVS mechanism will be based on SV measurement. Therefore, we propose to include SV measurements as a standard tool for diagnosing VVS, along with ICG and blood pressure monitoring. This conclusion is in line with our previous analysis based on other entropy measures [17,39,40,41]. Others investigators arrived at similar conclusions based on the measurement of SV in the early tilt [42].

When comparing TE between the HUTT(+) and HUTT(-) groups in the consecutive phases of the test, we did not observe any significant differences between phase I and IIa (Table 3). This lack of differences indicates that TE is not useful as a tilt test outcome predictor in supine position or in early tilt. We also investigated changes of TE(driver-target) in different HUTT phases.

For RRI driver:

- in the HUTT(-) group there were significant differences between the following phases: supine vs. pre-syncope (I vs. III) and tilt vs. pre-syncope (IIa vs. III) for each target (TE(RRI-sBP), TE(RRI-dBP) and TE(RRI-SV): *p* < 0.05).
- Similar outcome was noted in HUTT(+) patients for TE(RRI-dBP). Significant differences between supine and pre-syncope were also observed for TE(RRI-sBP) (Figure 2a).

For sBP driver:

- we did not observe significant differences in both groups between HUT phases (Figure 2b).

For the driver dBP:

- statistically significant differences were noted only in the HUTT(-) group for TE(dBP-sBP). The value of TE(dBP-sBP) in phase IIa (tilt) increased significantly in comparison with phase I (supine) from 0.08 to 0.18. There were also significant differences between supine and pre-syncope (I vs. II).
- In HUTT(-) patients there were no significant differences (Figure 2c).

For the driver SV:

- In both groups the values of TE(SV-RRI), TE(SV-sBP), TE(SV-dBP) significantly increased between tilt and pre-syncope (IIa vs. III). The occurrence of syncope after the tilt is associated with the increasing information transfer from SV to all components of the observed system.
- In the HUTT(-) group the significant increasing of TE(SV-sBP) and TE(SV-dBP) was observed between the ntg administration and pre-syncope (IIb vs. III).
- In both groups values of TE(SV-sBP) and TE(SV-dBP) were significantly higher between supine and pre-syncope. The values of TE changed approximately by about 0.1 (Table 2 and Table 3).
- In pre-syncope phase for the HUTT(+) group the values of TE(SV-sBP) and TE(SV-dBP) were: 0.19 and 0.17 and correspondingly in the HUTT(-) group 0.18 and 0.17 (Table 2 and Table 3).

In both groups the information transfer in the pre-syncope phase was at the same level. We suppose that such proportions of TE between SV and blood pressure are necessary for syncope to occur and in the case of the HUTT(-) group ntg administration allows to increase the TE value to a similar level as one seen in HUTT(+) patients in the pre-syncope phase. At this stage it is only our suggestion and this phenomenon requires further and more extensive investigation. In both groups values of TE(SV-sBP) and TE(SV-dBP) were significantly higher between supine and pre-syncope. The range of changes of TE was approximately 0.1 (Table 2 and Table 3).

The above observations confirmed our previous conclusion, that SV is a parameter which should be included in the further research focused on the mechanism of vasovagal syndrome occurrence and as such should be included in the diagnostic procedure as a standard measurement.

## 5. Limitations

We are aware that the analyzed hemodynamic parameters and their complexity depend on age and gender. Due to the small size of our study group we were not able to analyze the impact of age and gender in our investigation. We also did not investigate the impact of antihypertensive drugs which were not withdrawn in our group of patients, but we have not got enough data to perform additional analyses because only a few patients continued their treatment regime with antihypertensive drugs.

## 6. Conclusions

The information transfer measured with TE showed that in consecutive phases of HUTT, the driver system changes from RRI in supine position to sBP in upright position and finally to SV in the pre-syncope phase. The information transfer measured with TE is not useful in VVS prediction in the early phases of the test. We also did not observe significant differences in the information transfer related with baroreflex regulation. We suppose that this observation and the significant changes in TE for SV as a driver are the factors that will contribute to better understanding of the mechanism of vasovagal syndrome. This suggestion requires further investigation, with subjects’ gender and age taken into account and comparison of the results with those of healthy subjects.

## Figures and Tables

**Table 1:** The baseline characteristics of the study group. The parameters: age, sBP, dBP and HR are presented as (mean ± std). The other parameters are number of patients.

Baseline	HUTT(+) (*n* = 57: F = 43, M = 14)	HUTT(-) (*n* = 23: F = 17, M = 6)
Female-age [y]	35.6 ± 16	32.3 ± 12
Male-age [y]	41.7 ± 15.6	43 ± 15
HR [bpm]	72.06 ± 9.98	66.1 ± 16.8
sBP [mmHg]	108.6 ± 25.4	102.6 ± 20.4
dBP [mmHg]	68.0 ± 19.7	66.1 ± 16.8
Hypertension	3	0
Diabetes	0	0
Medication	2	4

**Table 2:** Descriptive statistics (mean ± std) and median [IQR] of TE (driver-target) in consecutive phases (I, IIa and III) of tilt test for the HUTT (+) group.

Transfer Entropy TE (Driver-Target)	Phase I (Supine)	Phase IIa (Tilt)	Phase III (Pre-Syncope)
	mean ± std median [IQR]	mean ± std median [IQR]	mean ± std median [IQR]
TE (RRI-sBP)	0.14 ± 0.12	0.13 ± 0.09	0.07 ± 0.08
	0.10 [0.13]	0.11 [0.10]	0.04 [0.08]
TE (RRI-dBP)	0.20 ± 0.18	0.16 ± 0.11	0.09 ± 0.09
	0.15 [0.21]	0.15 [0.13]	0.07 [0.09]
TE(RRI-SV)	0.17 ± 0.15	0.13 ± 0.12	0.11 ± 0.10
	0.15 [0.20]	0.10 [0.12]	0.07 [0.12]
TE (sBP-RRI)	0.08 ± 0.10	0.10 ± 0.10	0.10 ± 0.13
	0.15 [0.08]	0.07 [0.16]	0.05 [0.12]
TE (sBP-dBP)	0.07 ± 0.10	0.10 ± 0.12	0.07 ± 0.09
	0.03 [0.20]	0.07 [0.18]	0.05 [0.10]
TE(sBP-SV)	0.09 ± 0.11	0.09 ± 0.09	0.09 ± 0.10
	0.06 [0.09]	0.08 [0.09]	0.07 [0.14]
TE (dBP-RRI)	0.04 ± 0.05	0.08 ± 0.09	0.06 ± 0.08
	0.03 [0.20]	0.06 [0.13]	0.03 [0.08]
TE(dBP-sBP)	0.09 ± 0.11	0.19 ± 0.19	0.15 ± 0.15
	0.05 [0.08]	0.13 [0.23]	0.11 [0.17]
TE(dBP-SV)	0.04 ± 0.06	0.05 ± 0.05	0.09 ± 0.10
	0.02 [0.05]	0.03 [0.08]	0.04 [0.06]
TE (SV-RRI)	0.12 ± 0.13	0.08 ± 0.10	0.12 ± 0.11
	0.09 [0.15]	0.05 [0.12]	0.10 [0.12]
TE (SV-sBP)	0.08 ± 0.09	0.05 ± 0.07	0.19 ± 0.12
	0.05 [0.07]	0.02 [0.07]	0.18 [0.17]
TE (SV-dBP)	0.07 ± 0.10	0.07 ± 0.08	0.17 ± 0.13
	0.06 [0.13]	0.04 [0.11]	0.14 [0.19]

**Table 3:** Descriptive statistics (mean ± std) and median [IQR] of TE (driver-target) in consecutive phases (I, IIa and III) of tilt test for the HUTT(-) group.

Transfer Entropy TE (driver-target)	Phase I (Supine)	Phase IIa (Tilt)	Phase IIb (Nt)	Phase III (Pre-Syncope)
	mean ± std median [IQR]	mean ± std median [IQR]	mean ± std median [IQR]	mean ± std median [IQR]
TE (RRI-sBP)	0.14 ± 0.12	0.15 ± 0.08	0.07 ± 0.06	0.05 ± 0.05
	0.09 [0.17]	0.09 [0.17]	0.07 [0.08]	0.04 [0.07]
TE (RRI-dBP)	0.25 ± 0.22	0.18 ± 0.12	0.08 ± 0.09	0.07 ± 0.08
	0.18 [0.26]	0.16 [0.16]	0.05 [0.09]	0.05 [0.08]
TE(RRI-SV)	0.20 ± 0.18	0.14 ± 0.12	0.12 ± 0.14	0.06 ± 0.06
	0.09 [0.17]	0.09 [0.17]	0.07 [0.08]	0.04 [0.07]
TE (sBP-RRI)	0.09 ± 0.11	0.09 ± 0.09	0.07 ± 0.08	0.09 ± 0.08
	0.05 [0.07]	0.07 [0.14]	0.04 [0.10]	0.07 [0.17]
TE (sBP-dBP)	0.07 ± 0.08	0.10 ± 0.11	0.05 ± 0.07	0.11 ± 0.11
	0.06 [0.12]	0.07 [0.20]	0.07 [0.08]	0.10 [0.10]
TE(sBP-SV)	0.10 ± 0.11	0.06± 0.06	0.10± 0.09	0.05 ± 0.05
	0.09 [0.11]	0.06 [0.06]	0.07 [0.08]	0.08 [0.12]
TE (dBP-RRI)	0.03 ± 0.04	0.11 ± 0.10	0.09 ± 0.10	0.06 ± 0.09
	0.02 [0.06]	0.06 [0.13]	0.07 [0.08]	0.03 [0.08]
TE (dBP-sBP)	0.09 ± 0.13	0.12 ± 0.14	0.13 ± 0.15	0.10 ± 0.12
	0.05 [0.08]	0.13 [0.23]	0.08 [0.13]	0.11 [0.17]
TE(dBP-SV)	0.04 ± 0.08	0.06± 0.08	0.09 ± 0.09	0.05 ± 0.05
	0.02 [0.05]	0.03 [0.08]	0.06 [0.15]	0.04 [0.06]
TE (SV-RRI)	0.09 ± 0.08	0.08± 0.12	0.13 ± 0.08	0.16±0.12
	0.06 [0.16]	0.02 [0.16]	0.13 [0.12]	0.13 [0.19]
TE (SV-sBP)	0.06 ± 0.04	0.06± 0.06	0.14 ± 0.11	0.18±0.10
	0.06 [0.10]	0.04 [0.13]	0.22 [0.17]	0.16 [0.16]
TE (SV-dBP)	0.09 ± 0.09	0.08 ± 0.09	0.17 ±0.09	0.17±0.12
	0.04 [0.17]	0.04 [0.12]	0.17 [0.14]	0.13 [0.18]

**Table 4:** Results of Mann–Whitney test (with Bonfferioni correction) for comparisons of TE (driver-target) between HUTT(−) and HUTT(+) groups in phase I, IIa and III of HUTT. ↑ and ↓ indicate respectively a higher and lower value of TE in the HUTT(−) group in comparison with the HUTT(+) group.

HUTT(-) vs. HUTT(+)						
TE(Driver-Target)	Phase I (Supine)	*Adjusted* *p*	Phase IIa (Tilt)	*Adjusted* *p*	Phase III (Pre-Syncope)	*Adjusted* *p*
TE (RRI-sBP)	↑	1	↑	1	↑	1
TE (RRI-dBP)	↑	1	↑	1	↓	1
TE(RRI-SV)	↑	1	↑	1	↓	0.135
TE (sBP-RRI)	↑	1	↓	1	↓	1
TE (sBP-dBP)	↑	1	↑	1	↑	0.27
TE(sBP-SV)	↑	1	↓	1	↑	1
TE (dBP-RRI)	↓	1	↑	0.87	↓	1
TE(dBP-sBP)	↓	1	↓	0.27	↓	0.8
TE(dBP-SV)	↓	1	↑	1	↓	1

TE (SV-RRI)	↓	1	↓	0.87	↑	0.57
TE (SV-sBP)	↓	1	↑	1	↓	1
TE (SV-dBP)	↓	1	↑	1	↓	1

## Abbreviations

TLOC	transient loss of consciousness
TE	transfer entropy
TE (driver-target)	transfer entropy form driver to target
VVS	Vasovagal Syndrome
HUTT	head up tilt test
HUTT(+)	patients with syncope during the passive phase of HUTT
HUTT(−)	patients without syncope during the passive phase of HUTT, requiring pharmacological provocation with nitroglycerine (ntg)
RRI	R-R intervals
sBP	systolic blood pressure
dBP	diastolic blood pressure
SV	stroke volume
